# Analysis of particles from hamster lungs following pulmonary talc exposures: implications for pathogenicity

**DOI:** 10.1186/s12989-020-00356-0

**Published:** 2020-06-04

**Authors:** Erika Sato, Sandra A. McDonald, Yuwei Fan, Shaina Peterson, Joseph D. Brain, John J. Godleski

**Affiliations:** 1 Penn State DuBois, Pennsylvania State University, Dubois, PA 15801 USA; 2 John J. Godleski MD PLLC, 304 Central Ave, Milton, MA 02186 USA; 3 Goldman School of Dental Medicine, Boston University, Boston, MA 02118 USA; 4 Department of Environmental Health, Harvard T.H. Chan School of Public Health, Boston, MA 02115 USA; 5 Department of Pathology, Harvard Medical School, Boston, MA 02115 USA

**Keywords:** Talc, Granite, Fibers, Dust, Pulmonary toxicity, Hamster, Scanning electron microscopy, Inflammation, Lungs

## Abstract

**Background:**

Talc, a hydrous magnesium silicate, often used for genital hygiene purposes, is associated with ovarian carcinoma in case-control studies. Its potential to cause inflammation, injury, and functional changes in cells has been described. A complication of such studies is that talc preparations may be contaminated with other materials. A previous study by (Beck et al. Toxicol Appl Pharmacol 87:222-34, 1987) used a hamster model to study talc and granite dust exposure effects on various biochemical and cellular inflammatory markers. Our current study accessed key materials used in that 1987 study; we re-analyzed the original talc dust with contemporary scanning electron microscopy and energy dispersive x-ray analysis (SEM/EDX) for contaminants. We also examined the original bronchoalveolar lavage (BAL) cells with polarized light microscopy to quantify cell-associated birefringent particles to gain insight into the talc used.

**Results:**

SEM/EDX analyses showed that asbestos fibers, quartz, and toxic metal particulates were below the limits of detection in the original talc powder. However, fibers with aspect ratios ≥3:1 accounted for 22% of instilled material, mostly as fibrous talc. Talc (based on Mg/Si atomic weight % ratio) was the most abundant chemical signature, and magnesium silicates with various other elements made up the remainder. BAL cell counts confirmed the presence of acute inflammation, which followed intratracheal instillation. Measurements of cell associated birefringent particles phagocytosis revealed significant differences among talc, granite, and control exposures with high initial uptake of talc compared to granite, but over the 14-day experiment, talc phagocytosis by lavaged cells was significantly less than that of granite. Phagocytosis of talc fibers by macrophages was observed, and birefringent particles were found in macrophages, neutrophils, and multinucleate giant cells in lavaged cells from talc-exposed animals.

**Conclusion:**

Our data support the contention that talc, even without asbestos and other known toxic contaminants, may elicit inflammation and contribute to lung disease. Our findings support the conclusions of (Beck et al. Toxicol Appl Pharmacol 87:222-34, 1987) study. By analyzing particulate exposures with polarized light microscopy and SEM/EDX, fibrous talc was identified and a distinctive pattern of impaired particulate ingestion was demonstrated.

## Introduction

Talc, a hydrous magnesium silicate that is commonly used for genital hygiene purposes, has been associated with ovarian carcinoma in multiple case-control studies [1–5]. In addition, it has been known for decades that significant occupational exposure to talc is associated with lung disease [6, 7] and inflammation [8, 9]. The assessment of talc’s pathogenic effects may sometimes be complicated when other materials contaminate talc preparations. Moreover, talc exists in both fibrous and non-fibrous forms. The pathogenic potential of a given respirable toxic dust may be influenced by the proportion and composition of fibers. We define fibers as having roughly parallel sides and a length/width ratio of 3:1 or greater. Such fibers, with diameters of no more than a few microns, may penetrate deep into the lungs and deposit in both large and small airways as well as alveoli when inhaled, even with fiber length > 10 μm. The Stanton hypothesis states that for the same mass, longer fibers have a higher pathogenic potential than shorter ones [10]. Fibers whose association with lung disease are well known include serpentine and amphibole asbestos [11]. Talc preparations from approximately 1970 onward have been thought to be much less likely to contain asbestos [12], since that was approximately when talc manufacturers claimed to voluntarily use asbestos-free talc (thus creating a cosmetic grade “free of asbestos” vs. industrial grade which may have asbestos) [13, 14]. However, talc may contain other substances, including other silicates that may be in fibrous form. Therefore, when assessing toxic potential, it is important to measure both chemical compositions *and* the proportion of the dust that is fibrous.

This current study expands on the earlier study by Beck et al. [15], using the same materials still available from that 1987 study. In that study, the investigators used an animal bioassay to better understand exposure-dose relationships and relative pathogenicity caused by talc and granite workplace exposures. Granite is a heterogeneous mineral that usually contains micas, clay, feldspar, and quartz, which include silicon (Si) along with magnesium (Mg), aluminum, calcium, iron, potassium, sodium, and others; the silicon component can account for 20–30% of the composition and is in the form of quartz (silicon dioxide/silica). Granite was studied here in relation to talc. In the past, granite dust inhalation resulted in workers developing high rates of silicosis and premature deaths from tuberculosis [16]. By using conditions in their hamster bioassay designed to simulate inhalation exposures, Beck et al. [15] aimed to compare the harmful effects of talc and granite dusts. The granite dust, given the historical data on its workplace exposures, was used by the group as a positive control. Large amounts of respirable talc and granite dust were collected from typical workplace environments using a high-volume air sampler. They assessed the silica content of the talc dust and found a quartz content < 1%. Scanning electron microscopy (SEM) was used to determine the proportion of fibers in the dusts, and they reported that no fibers were identified [15]. Talc dust was sonicated in physiologic saline and the resulting dust suspension was intratracheally instilled into the hamster lungs. There were at least 6 hamsters used in each exposure group, and various doses ranging up to 3.75-mg/100 g bodyweight were used.

Beck et al. [15] reported size characteristics for the talc and granite dust particles (important since particle size has respiratory and pathogenic implications). The talc particles were consistently larger than the granite particles across various measurement methods. The count median diameters of the talc and granite particles were 0.8 μm and 0.55 μm, and the mass median aerodynamic diameters for the talc and granite particles were 7.5 μm and 1.9 μm, respectively.

Beck et al. [15] also assessed the time course of lung responses at 1, 4, 7, and 14 days following a single intratracheal instillation. The animals were sacrificed and bronchioalveolar lavage (BAL) fluid samples were obtained and analyzed. Lactate dehydrogenase, *β-N-*acetylglucosaminidase, and albumin levels in the BAL were measured, and cells were numerically assessed in the cell pellet fraction of the BAL after centrifugation. A smear was made from the pellet and a cellular differentiation count was performed; macrophages were morphologically differentiated from neutrophils and other leukocytes. A separate group of hamsters was injected with instilled colloidal radioactive gold to measure pulmonary macrophage function. The gold-injected hamsters were sacrificed by exsanguination 90 min after the injection, then lung washing was performed and the cell count and a numerical parameter (lambda), defined as the fraction of gold colloid ingested by pulmonary macrophages during the 90-min incubation, was calculated.

The results of assays in the Beck et al. [15] study, listed in Table 1, demonstrated that talc and granite dusts can have significant pathogenic effects with increases in polymorphonuclear leukocytes, intra-alveolar hemorrhage, and increased biochemical markers of inflammation. Important effects on macrophage function as measured by the uptake of radioactive gold nanoparticles showed decrease phagocytic ability due to talc at the longer time points, which was not seen with granite. In general, both talc and granite dusts were found to cause significant lung injury in this study.

**Table 1:** Outcome data from tables and figures from Beck et al. 1987.*

Parameters		Day 1	Day 4	Day 7	Day 14
**BAL Cell Counts with 3.75 mg/100 g Body Weight Intra-tracheal (IT) Dose**					
Macrophages (Cell Number per total lavage volume, × 10^6^)	Talc	5.5 ± 0.3 (NS)	10 (NS)	8 (NS)	10 (S)
	Granite	6.7 ± 1.0 (S)	13 (S)	13 (S)	13 (S)
	Control	6.0 ± 0.5	6.5	6	6.2
Neutrophils (Cell Number per total lavage volume, ×10^6^)	Talc	11.7 ± 1.1 (S)	4 (S)	4 (S)	4 (S)
	Granite	20 ± 2.8 (S)	15 (S)	4 (S)	0.8 (S)
	Control	1.5 ± 0.6	0.3	0.1	0.2
**BAL fluid Analyses with 3.75 mg/100 g Body Weight IT Dose**					
Lactic Dehydrogenase (mU/ml Lavage fluid)	Talc	139 ± 15 (S)	80 (S)	65 (NS)	55 (S)
	Granite	125 ± 7 (S)	59 (S)	43 (S2)	37 (S2)
	Control	19 ± 1	22	25	21
Peroxidase (mU/ml Lavage fluid)	Talc	210 ± 37 (S)	N/A	N/A	N/A
	Granite	196 ± 36 (S)	N/A	N/A	N/A
	Control	2 ± 2 ± 0.3	N/A	N/A	N/A
ß-N-Acetyl Glucosaminidase (mU/ml Lavage fluid)	Talc	159 ± 9 (S)	80 (S)	82 (S)	84 (S)
	Granite	187 ± 21 (S)	40 (S)	35 (S)	30 (S5)
	Control	25 ± 6	26	23	28
Albumin (μg/ml Lavage Fluid)	Talc	1380 ± 116 (S)	N/A	N/A	N/A
	Granite	2130 ± 262 (S)	N/A	N/A	N/A
	Control	66 ± 4	N/A	N/A	N/A
**In vivo Phagocytosis: Lambda assay uptake of labeled Particles after IT dust (3.75 mg/100 g Body Wt Dose)**					
Lambda: Fraction of Radioactive Gold Particles Phagocytized	Talc	0.46 ± 0.07 (S)	0.56 (S)	0.54 (S5)	0.52 (S2)
	Granite	0.47 ± 0.10 (S)	0.85 (S5)	0.82 (NS)	0.7 (NS)
	Control	0.66 ± 0.3	0.75	0.73	0.78

* All data from figures are estimates of the means. When these data were also present in tables, the estimates were never more than 2% different from the numbers available in the tables; table data are shown if both were available. All data with distributions are shown as Mean ± SEM. N/A indicates data were not available from the Beck et al. paper. All statistical analyses are shown as *p* value of difference from control (C). NS indicates not significantly different from control. S indicates significantly different from control, S1p < 0.01 vs control. S2, *p* < 0.02 vs control, S5 p < 0.05 vs control

The Beck et al. [15] study had limited details on the talc powder characterization, especially with regard to chemical composition, mineralogical characteristics, and fiber content. Polarized light microscopy was not used in the original study to assess cell-associated particles. The purpose of this current study was to independently assess the Beck study’s previous cellular assay findings from the archived BAL slides using birefringence under polarized light (a characteristic of talc and certain other minerals, especially silicates) to identify particles associated with macrophages and neutrophils. We also looked for the presence of birefringent fiber ingestion by the cells. We aimed to further characterize the talc dust exposure used in that study, especially to assess the presence and composition of any 1) fibers, whether asbestos or not, and 2) toxic metals or other substances that could enhance pathogenicity. Our goal was a better understanding of the degree to which talc can induce pulmonary inflammation and cellular functional deficits on its own, as opposed to other fibrous or toxic components that may contribute to pathogenicity.

## Materials and methods

Beck et al. [15] used four animals for each experimental group and three animals for the control in individual experiments, which were repeated at least once. Only male Syrian Golden Hamsters were used in these studies. The animals were exposed to different doses of talc per 100 g bodyweight. For the current study, the BAL slides from the 3.75 mg/100 g body weight dose were studied. These slides had been preserved since the original study, and were reanalyzed at high-power (400x) magnification on a BH-2 Olympus light microscope with polarizing light and photomicrographic capabilities. Counts of macrophages, neutrophils, lymphocytes, and giant cells were recorded using 10 random microscopic fields per slide. In each field, the number of birefringent particles was assessed, including both particles and fibers. The number of particles associated with cells was counted and all particles in a field were assessed by size (categorized as either < 6 μm or ≥ 6 μm in greatest dimension) and aspect ratio ≥ 3:1 for fibers). Macrophages and neutrophils were scored as having either > 3, or 3 or less, birefringent cell-associated particles. Each field was examined for fibers and the number recorded. Each slide, representing talc, granite, or control exposure, at each time point, was reviewed. Particles were counted as cell-associated if they were visualized with the cytoplasm of the cell. If the particles were only attached to the edge of the cell, they were not counted. For particles in or overlying the cytoplasm to be counted, they needed to be in the same plane of focus with cell organelles. This criteria eliminated any particles that might be overlying or beneath the cell in the mounting medium on a slide, but did not permit distinction between internalized versus attached particles. Data comparing the number of macrophages with controls, as well as neutrophil numbers to controls, were created. The number of fibers and giant cells were recorded separately.

To characterize the dust used for exposure, we utilized bulk samples of the talc powder, which had been used in the original Beck at al [15]. study and had been preserved in a secure particle sample safe in the laboratory of author JDB. Thus, the dust samples we analyzed were from the same batch as for the BAL slides from 1987. A talc sample was analyzed with a Hitachi SU6600 field emission SEM with an Oxford energy dispersive X-ray analysis system (EDX) (Oxford Instrument software was Aztec 3.3; EDX detector model was X-Max 50 SDD). To prepare the samples, the talc powder was mixed into prefiltered distilled water and placed into two separate test tubes and each sonicated using a Branson probe sonicator model 200 at maximum power for 30 s. The first sample, labeled Sample A, contained 10 mL of particle suspension. The second sample, labeled Sample B, contained 3 mL of particle suspension. Thus, Sample B was meant to represent approximately one-third of Sample A, in order to provide a range of sample density for SEM/EDX examination. The two test tubes were then individually filtered using 13 mm, 0.22 μm pore Whatman filters (product # 110406) to disperse particles onto the filter. Sample A was found to have optimal dispersion of particulate for analysis by SEM/EDX, counting and analyzing the first 200 particles or fibers that were encountered in a succession of random 2000x scanning fields.

Data were analyzed using analysis of variance with Tukey’s post-hoc test to assess statistical significance among multiple treatment groups at all time points assessed. Quantitative data shown in Figs. 3, 4, 5, 6 are presented as mean ± standard error of the mean (SE).

## Results

**Fig. 1:**
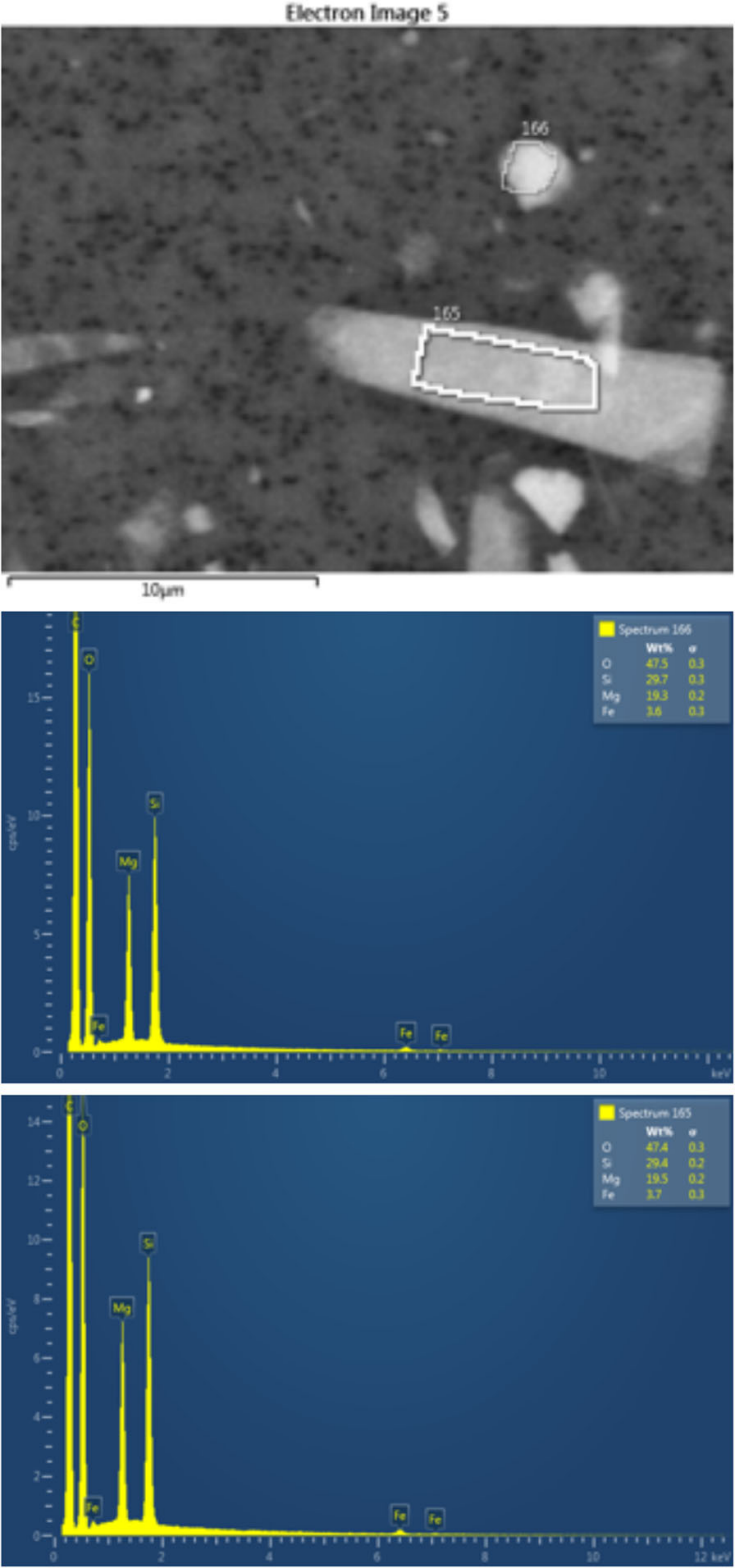
SEM morphology of two nearby particles, one in particle form and the other with the appearance of a fiber (aspect ratio 5:1). Both are typical of talc by quantitative EDX

SEM/EDX analysis data for 200 particles from the talc sample are in Table 2. All particles were found to be magnesium (Mg) silicates. 78% of the material instilled was in the form of non-fibrous particles with aspect ratios < 2.0, but 22% were classified as fibers based on the dual criteria of aspect ratio ≥ 3:1 and approximately parallel sides. 68% of all identified fibers were fibrous talc; no fibers had the elemental compositions required for any asbestos fiber type. The majority had elemental weight % ratios characteristic of talc. Figure 1 shows a representative example of two EDX spectra of talc particles, one non-fibrous and the other a fiber with an aspect ratio of ~ 5:1.

**Table 2:** Characteristics of particles and fibers by SEM/EDX

Particles or Fibers	Count	Percentage	Greatest Dimension - Length (μm) Ave ± SD	Aspect Ratio Ave
Particles by Morphology	156	78.0%	7.3 **±** 5.0	1.7
Fiber by Morphology Aspect ratio 3:1or >	44	22.0%	13.1 **±** 9.3	6.3
Asbestos by EDS	0	0%	0	0
**Compositional Analysis**				
**Talc by EDX MgSi wt% ratio 0.595–0.695**	**107**	**53.5%**		
Particles	77	72.0%	9.2 ± 5.4	1.8
Fibers - Aspect ratio 3:1or >	30	28.7%	14.9 ± 9.8	6.7
**MgSi by EDX but not talc by wt% ratio**	**5**	**2.5%**		
Particles	3	60.0%	10.8 ± 6.0	1.4
Fibers - Aspect ratio 3:1 or >	2	40.0%	7.9 ± 5.9	4.1
**MgSi by EDX, but not talc by composition**	**60**	**30.0%**		
Particles	51	85.0%	5.3 ± 4.0	1.7
Fibers - Aspect ratio 3:1or >	9	15.0%	8.8 ± 6.3	6.3
**Mg + Si + > 5% Ti, Al, or Ca**	**27**	**13.5%**		
Particles	24	88.9%	5.5 ± 4.5	1.5
Fibers - Aspect ratio 3:1or >	3	11.1%	11.7 ± 10.5	4.8
**Mg + Si + > 5% Cr** (particle)	**1**	**0.5%**	2.03	1.2

Magnesium silicates containing various other elements, present in amounts of < 5% based on quantitative peak ratios (i.e., atomic weight percent ratios) for EDX elemental composition made up approximately 30% of the particles/fibers. This category included iron, aluminum, calcium, and titanium. Potentially toxic elements (including nickel and vanadium) were occasionally detected at a trace level, but were never at a level > 1% (as measured by EDX composition), except for a single magnesium silicate particle that was found to have > 5% chromium. Twenty-seven [17] magnesium silicate particles had other elemental components at concentrations > 5%, including: titanium, aluminum, and calcium. We found no particles with only the elements silicon (Si) and oxygen (O) (a signature consistent with quartz).

**Fig. 2:**
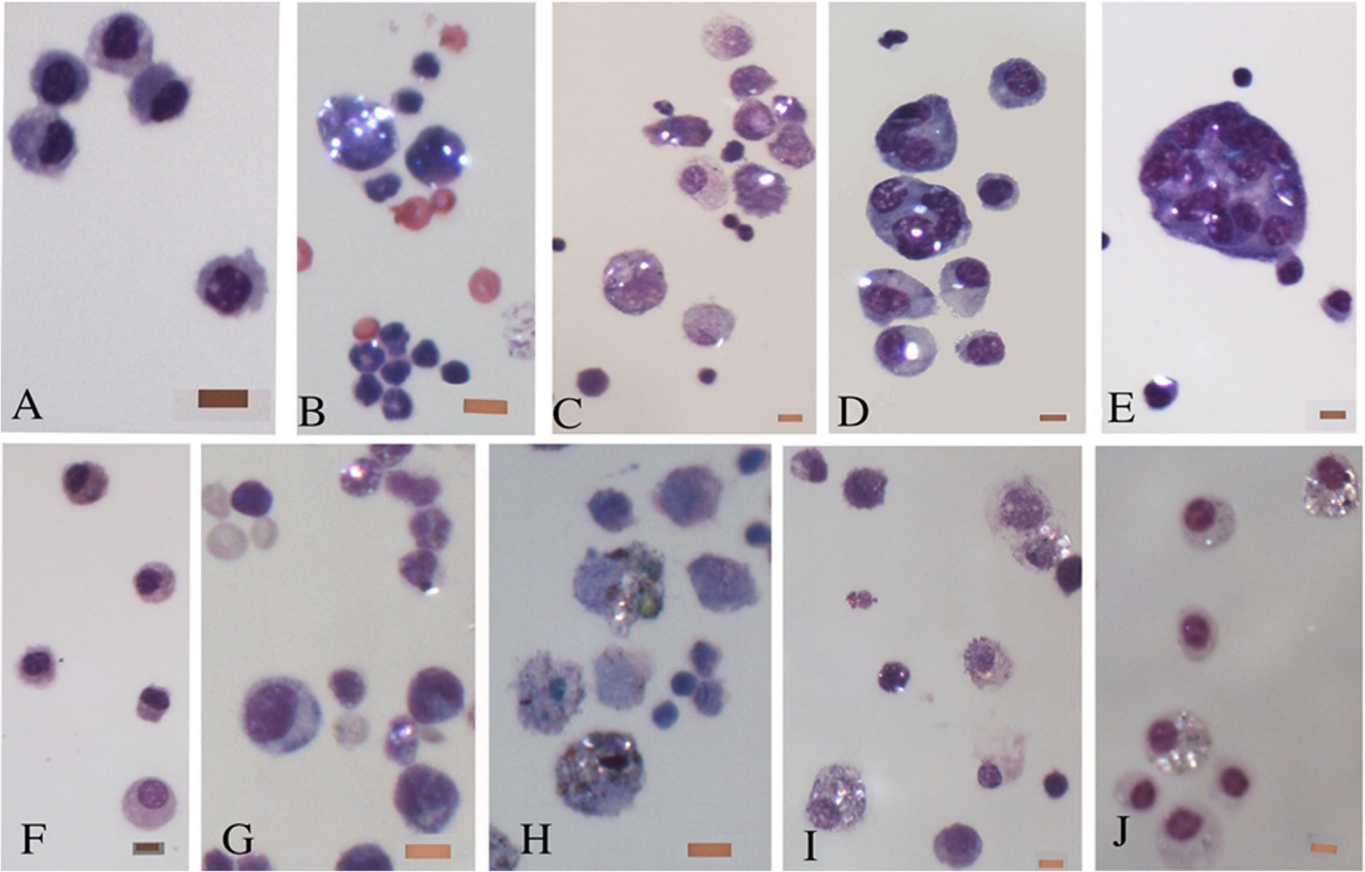
Photomicrographs of representative fields of BAL Giemsa stained slides from each time period and 2.5% treatment groups. All photomicrographs taken at 400X. Bar on each image represents 10 m. **a**. Control group for talc instillation studies (saline plus surface active material instillation). Mostly alveolar macrophages were observed in these slides. **b**. One day after 2.5% talc particle instillation, uptake of varying sized birefringent particles by macrophages can be observed as well as neutrophils and red cells in this field. **c**. Four days after talc instillation, birefringent particles are visible in both macrophages and neutrophils; lymphocytes are also visible in the field. **d**. Day 7 after talc instillation, multinucleate cells are seen as well as macrophages with single and multiple birefringent particles. **e**. Day 14 after talc instillation, true multinucleate giant cells are seen with numerous birefringent particles. Also, a fiber is observed in this giant cell with a 3:1 aspect ratio. **f**. Control group for granite instillation studies, again mostly macrophages were observed. **g**. One day after 2.5% granite particle instillation, uptake of both black colored particles and birefringent particles by macrophages can be observed but these are not as prominent as seen at this time point with talc as well as neutrophils and red cells in this field. **h**. Four days after granite instillation, both black and birefringent particles are visible in macrophages; lymphocytes are also visible in the field. **i**. Day 7 after granite instillation, macrophages and neutrophils with single and multiple birefringent particles are seen. **j**. Day 14 after granite instillation, no multinucleate giant cells were seen with granite at any time point, only numerous macrophages with single and multiple black and birefringent particles. Also, no fibers were observed in any slides of the granite treatment groups

Examples of cells from each time period of the 2.5% exposure groups are illustrated in Fig. 2 with photomicrographs of representative fields of Giemsa-stained BAL slides. The control group for talc instillation studies (saline plus surface active material instillation) had mostly alveolar macrophages. One day after the 2.5% talc particle instillation, the ingestion of variably-sized birefringent particles by macrophages was observed, along with neutrophils and red cells. Four days after talc instillation, birefringent particles were visible in both macrophages and neutrophils. Lymphocytes were also visible. By day 7 after talc instillation, multinucleate cells were seen as well as macrophages with single and multiple birefringent particles. By day 14, true multinucleate giant cells were seen with numerous birefringent particles. Also, a fiber was observed in the illustrated giant cell, which had a 3:1 aspect ratio.

The cellular morphology of the granite groups is illustrated in Fig. 2. The controls for granite instillation studies primarily contained macrophages. One day after the 2.5% granite particle instillation, phagocytosis of both black-colored particles and birefringent particles by macrophages could be observed, but these particles were not as prominent as was seen at this time point with talc. Also, there were fewer neutrophils and red cells. Four days after granite instillation, both black and birefringent particles were visible in macrophages, and lymphocytes were also observed in the BAL. On day 7 after granite instillation, macrophages and neutrophils with single and multiple birefringent particles were seen. By day 14 after granite instillation, only numerous macrophages with single and multiple birefringent particles were observed. No multinucleate giant cells were seen with the granite-exposed group at any time point. No fibers were observed in any slides of the granite-exposed groups.

Table 3 quantifies the percentages of particles by size or in the form of fibers by each day of post-instillation. The majority of particles (> 90% at all time periods) in the talc-exposed group were <  6 μm, even though the material used had measured median and mean sizes greater than this. Particles ≥6 μm were uncommon at all time points, and ranged from 2.0–3.7%. These particles were often cell-associated, but most did not appear to be ingested. Fibers accounted for 2.5–5% at various time points in the talc-exposed group, even though the fiber percentage was higher in the material used for instillation. In the granite-exposed group, at each time point, the percentage of particles < 6 μm was at least 99%, and no fibers were observed.

**Table 3:** Measurements of birefringent particles or fibers found on BAL slides using polarized light microscopy shown as percentages of particles by greatest dimension (< 6 m or > 6 m) and all fibers having > 3:1 aspect ratio and parallel sides

	Talc		Days after instillation		Granite	
***< 6 μm***	***6 or > μm***	***Fibers***		***< 6 μm***	***6 or > μm***	***Fibers***
93%	3%	4%	1	100%	0	0
95%	2.5%	2.5%	4	100%	0	0
92%	3.7%	4.3%	7	99%	1%	0
93%	2%	5%	14	99%	1%	0

Note: Ten high power microscopic fields without artifacts were randomly chosen for analysis. Variations in the numbers of cells counted were because of different cell densities on the slides. Figs. 3 through 6 in the paper are presentations of various cell types with cell-associated birefringent particles

**Fig. 3:**
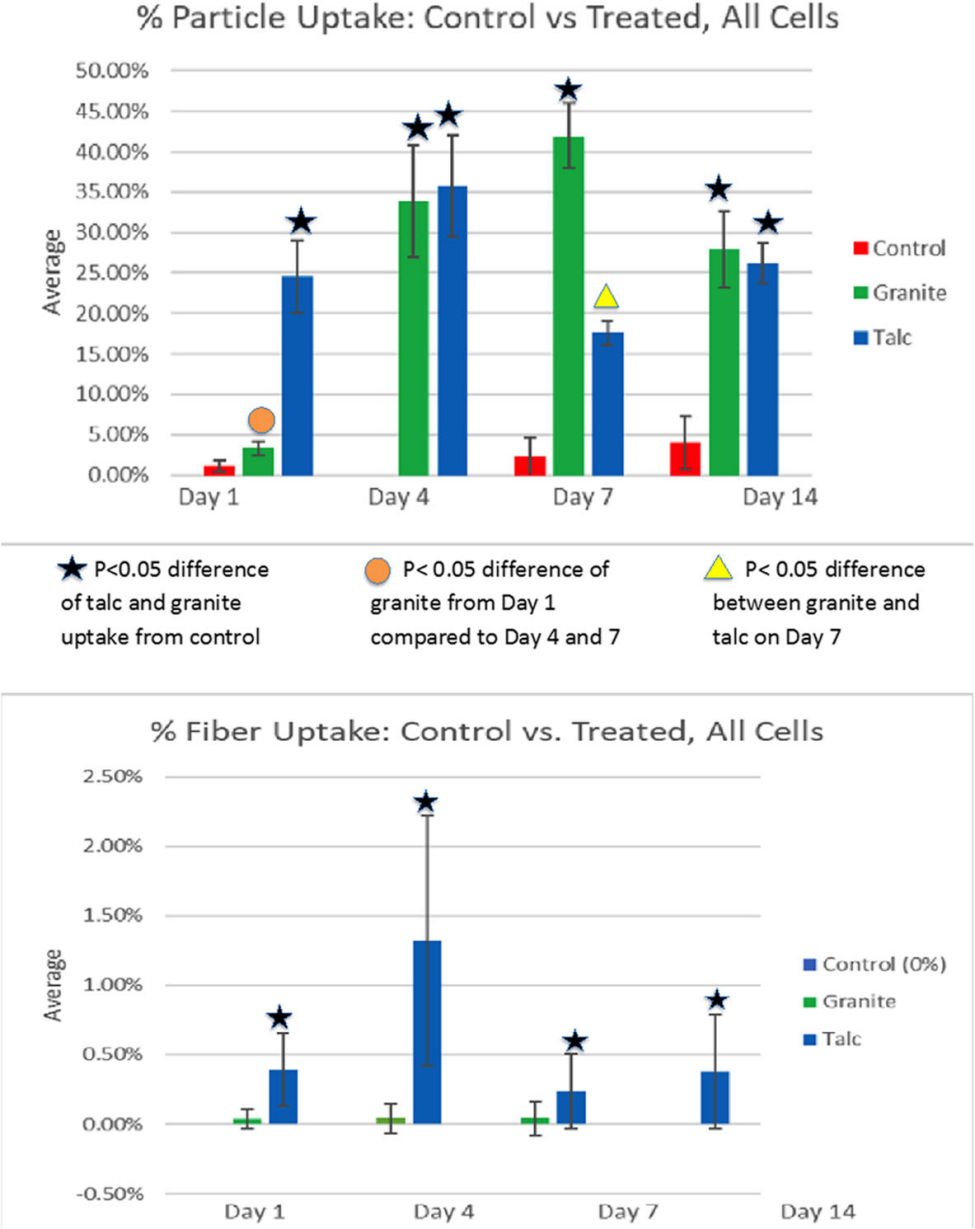
**a** Uptake of birefringent particles of the control group as well as the talc- and granite-exposed groups. Particle uptake was calculated by dividing the number of cells with birefringent particles by the total number of cells counted. The particle uptake ranged from 0% to roughly 40% depending on the day. Day 1 showed low levels of both the control and granite, but a high level of talc. Day 4 showed increased values of both talc and granite exposures. Day 7 showed a minimal change in the control. The granite, on day 7, continued to increase, while the talc decreased down to approximately 17%. Day 14 showed both a decrease in granite and an increase in talc to bring both groups to approximately 27%. The starred groups indicate the *p*-value difference of talc and granite was less than 0.05 from the control group. The circle was used when the *p*-value difference of granite was less than 0.05 from day 1 compared to day 4 and day 7. The square was used when the *p*-value difference between talc and granite was less than 0.05 on day 7. All data are shown as Mean ± SE. **b**. Percentage of cells with phagocytosed fibers in the BAL samples in the talc- and granite-exposed groups. The percentages were calculated by dividing the number of cells with fibers by the total number of cells counted. Each percentage was below 2%, showing the relative scarcity of cell-associated fibers compared with values for birefringent particles. Day 4 showed the highest number of fibers in the talc group at approximately 1.3%. The granite group had a nearly zero average throughout the 14 days. Compared to both the granite and control group, treated talc had the most amount of fibers. The star indicates the p-value difference of talc was less than 0.05 from the control and granite groups. All data are shown as Mean ± SE

**Fig. 4:**
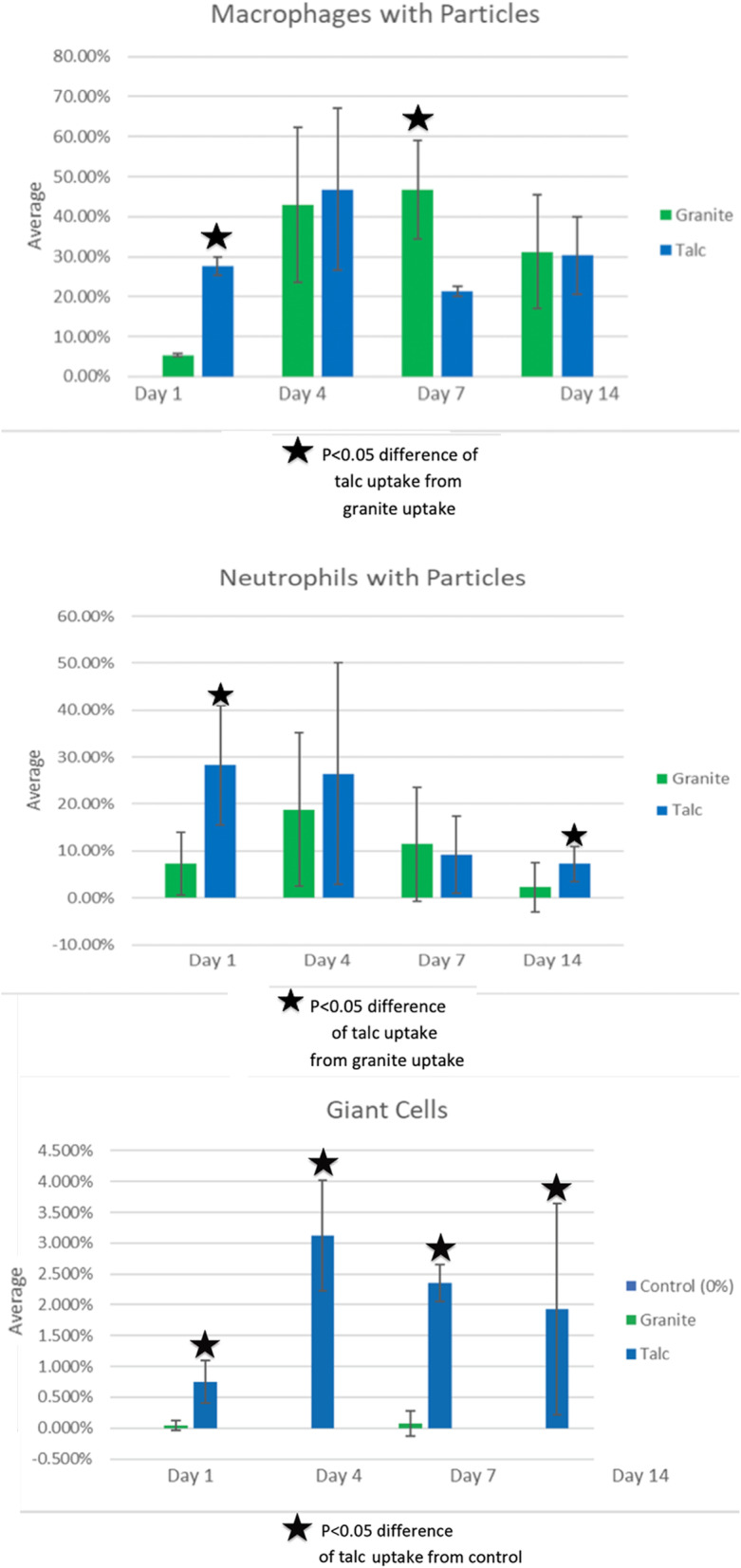
**a.** Percentage of cells with associated birefringent particles in macrophages from the talc-exposed or granite-exposed groups. The percentages were calculated by dividing the number of macrophages with particles by the total number of macrophages counted. Day 1 shows a low number of macrophages with particles for the granite group, but a higher level for the talc group. By day 4, both groups have increased with similar amounts approximate to 45%. Day 7 shows a large decrease in the number of macrophages in the talc group, but a slight increase in the number of macrophages in the granite group. By day 14, both have similar percentages at approximately 30%. The star indicates the p-value of the difference was less than 0.05 between the granite and talc groups. All data are shown as Mean ± SE. **b**. Percentage of cells with associated birefringent particles in the neutrophils of both the granite-exposed and talc-exposed groups. The percentages were calculated by dividing the number of neutrophils with birefringent particles by the total number of neutrophils counted. The granite group significantly increased from day 1 to day 4 and then proceeded to decrease. The talc group began high and then decreased over day 7 and day 14. The talc group had the highest average on day 1 at approximately 29%. The granite group had the highest average on day 4 with approximately 19%. Both groups had their lowest averages by day 14 with the talc group at approximately 3% and the granite group at approximately 7%. The star indicates the p-value of the difference between the granite and talc groups was less than 0.05. All data are shown as Mean ± SE. **c.** Percentage of giant cells in both the control group as well as the granite- and talc-exposed groups. The percentage was calculated by dividing the number of giant cells by the total number of cells counted. The talc–exposed group had a higher number of giant cells compared to the other two. The treated granite group had a minimal number of giant cells. The control group had 0% of giant cells. The p-value of the difference for talc-exposure versus each other group across each day was all less than 0.05. All data are shown as Mean ± SE

**Fig. 5:**
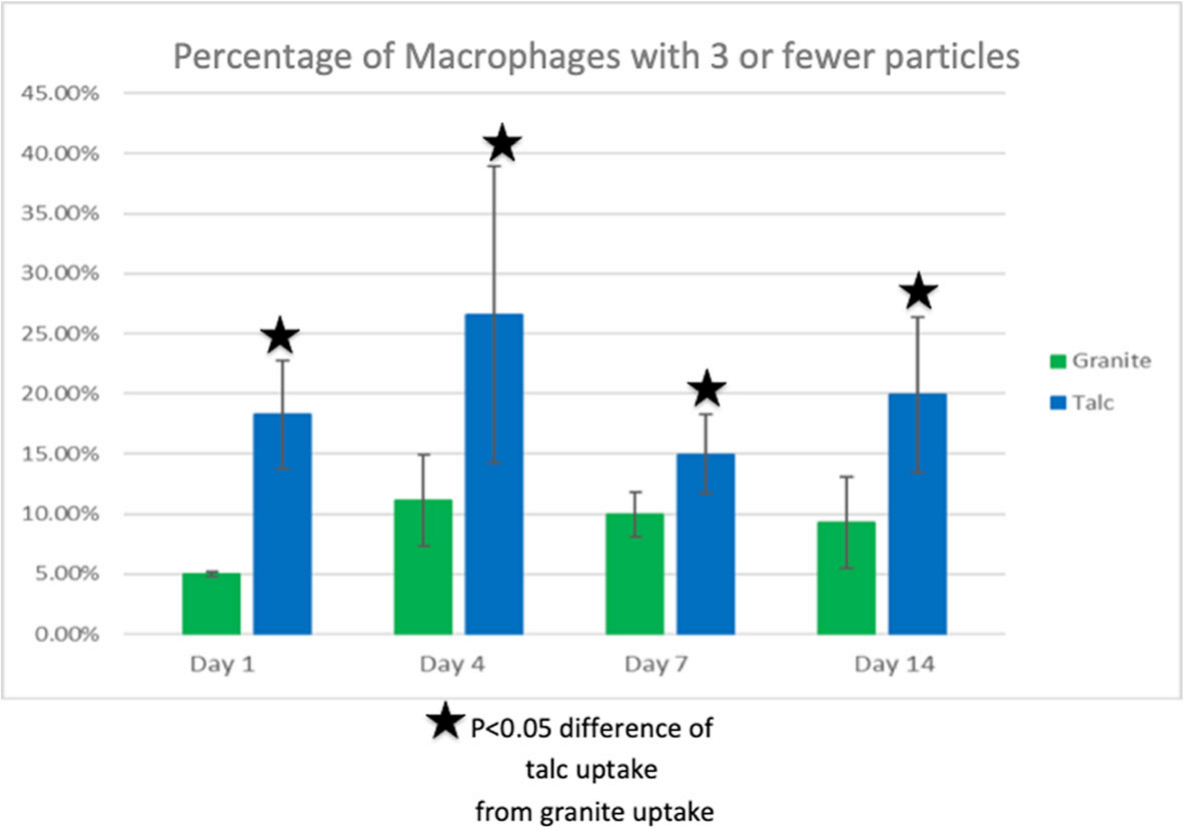
Percentage of macrophages with 3 or less cell-associated birefringent particles in the cells of granite- and talc-exposed groups. The percentages were calculated by dividing the number of macrophages with 3 or less birefringent particles by the total number of macrophages counted. There were higher values for talc-exposure across all the days compared to the granite group, and the overall difference as well as the difference on each day was statistically significant. All data are shown as Mean ± SE

**Fig. 6:**
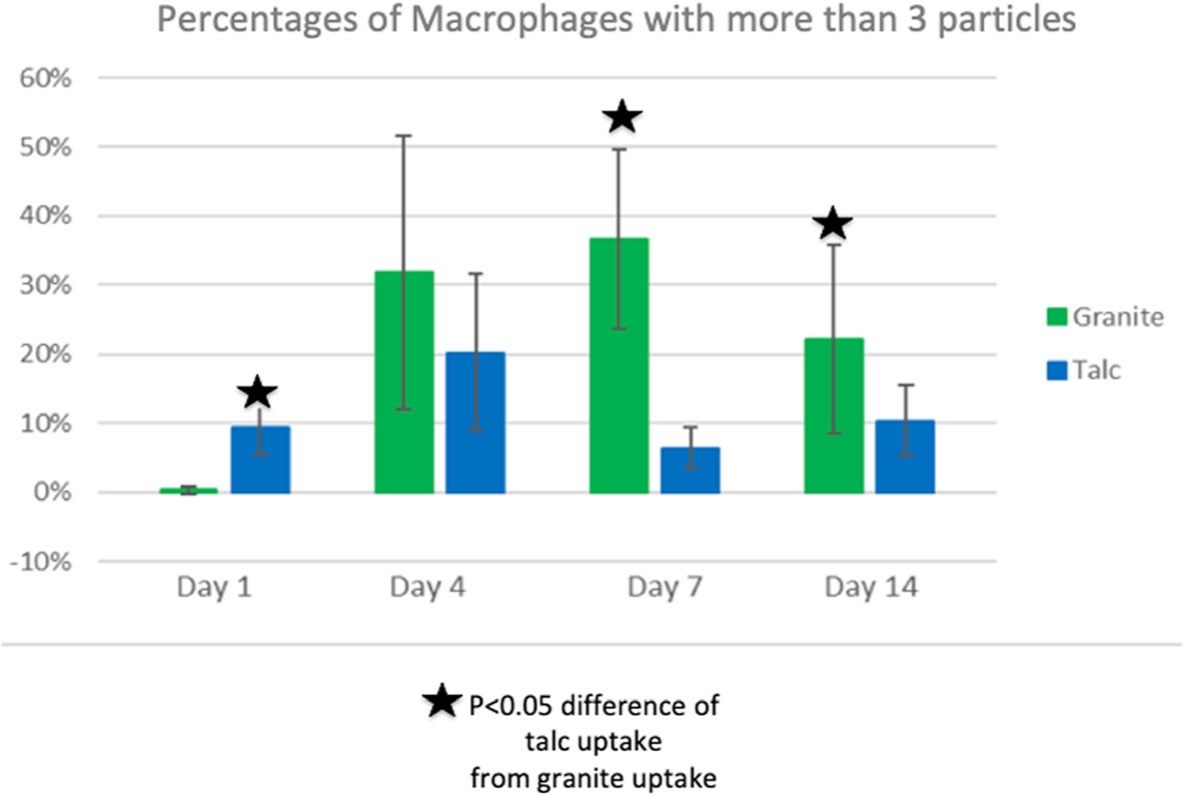
Percentage of macrophages with more than 3 cell-associated birefringent particles in the granite- and talc-exposed groups. On day 1, the granite group had approximately 1% of macrophages with more than 3 particles, while the talc group had approximately 10%. This was the only day where the talc group had a higher value compared to the granite group. The difference between talc- and granite-exposed groups on day 1 was statistically significant (*p* < 0.03). On day 4, the granite group increased to above 30%, while the talc group was approximately 20%, but this difference was not significant. On day 7, the granite group increased, while the talc group decreased below 10%, a highly significant difference (*p* = 0.0001). By day 14, the granite group decreased to approximately 22%, while the talc group increased to approximately 10% (p < 0.03). All data are shown as Mean ± SE

The percentages of cells with associated particles in the control group and talc- and granite-exposed groups are shown in Fig. 3a. (In Figs. 3, 4, 5, 6, all data are shown as percentages of cells with associated particles or fibers based upon assessment of the cells in 10 random fields per slide. Although this approach resulted in a variable number of cells counted per slide, the overall average number of cells assessed per slide was 327. Supplementary Table 1, lists the mean numbers of cells counted per slide assessed for the parameters shown in Figs. 3-6 for each treatment and time point assessment. The numbers range from 127 to 225 in saline controls, 255 to 576 in talc groups, and 225 to 771 in granite groups.) On day 1, the talc group showed the highest percentages of cells with associated particles, reaching nearly 25%, while both the control group and granite groups showed very few cells with associated particles. By day 4, as expected, there was no phagocytosis of birefringent particles in the control group, but the granite-exposed and talc-exposed groups had ingested particles with approximately 35% of all BAL cells of both groups having birefringent particles. By day 7, the number of cells with particles in the granite-exposed group continued to increase (now > 40%), while the talc-exposed group showed a decline in the percentage of macrophages with particles to < 20%. On day 14, the talc-exposed group and the granite-exposed group had just under 30% of the cells with ingested particles. The differences were statistically significant between the control and talc cellular ingestion percentages for days 1, 4, and 14, and for granite on days 4, 7, and 14. There was also a significant difference between cellular ingestion of granite on day 1 compared to day 4 and 7. There was a significant difference between the percentage of cells with cellular ingestion of talc and granite on day 7, when the % value for granite was more than twice that of talc.

Figure 3b shows the percentages of cells with ingested fibers of the control, granite-exposed, and talc-exposed groups over the 14 days. The control group consistently had 0% fibers throughout the 14 days, while the exposed granite group had a miniscule percentage. The most notable, though still low, percentages were from the talc-exposed group. None of the time points yielded more than 2% of cells with fiber phagocytosis in the exposed talc group, but the percentages of cells in the talc-exposure groups were statistically significantly higher at each time point than both the granite-exposed and control groups. Fibers in the cells of the talc-exposed group were highest on day 4. As shown in Fig. 2, some of the fiber phagocytosis involved multinucleate giant cells. Within the treated talc group, there was a significant difference in fiber levels between days 4 and 7 and between days 4 and 14. There was no significant difference between the control and the granite-exposed group.

Figure 4a shows the percentage of cell-associated particle ingestion in macrophages of both the talc- and granite-exposed groups. As expected, the particle phagocytosis by macrophages was very similar to the individual day patterns for all cell types, which can be appreciated when comparing Fig. 3a and Fig. 4a. On day 1, the percentage of cell-associated particles was ~ 5% for the granite group, while it was nearly 30% for the talc group. By day 4 and 7, the granite group had a higher percentage (approximately 45%), while the talc group was ~ 45% on day 4, but decreased to nearly 20% on day 7. On day 14, both the talc- and granite-exposed groups were similar (approximately 30%). Over the 14 days, the granite-exposed group increased from day 1 to 7, and then decreased from day 7 to day 14. The talc-exposed group increased from day 1 to 4, decreased from days 4 to 7, and then remained in the same range from day 7 to 14. Within the granite-exposed group, there were significant differences in the number of macrophages with particles between day 1 and day 4, and between day 1 and day 7. Within the talc-exposed group, there was only a significant difference in the percentage of macrophages with particles between day 4 and day 7. On the first day, the number of macrophages with particles was significantly different between the talc- and granite-exposed groups. There was also a significant difference between the two groups on day 7.

Figure 4b shows the percentage of cells exhibiting particle phagocytosis in polymorphonuclear neutrophil (PMN) leukocytes in the talc- and granite-exposed groups. This ingestion pattern of neutrophils with talc-exposure showed the greatest uptake of birefringent particles on days 1 and 4 with these being similar to macrophage uptake. However, the percentage of cells with birefringent particles was greater in macrophages on days 7 and 14 than by PMNs. On day 1, the granite-exposed group had a small percentage of cells with particle phagocytosis, while the talc-exposed group had almost 30%, a statistically significant difference. On day 4, the granite- and talc-exposed groups had similar percentages (near 20%.) By day 7, both percentages fell below 15%, with the granite group at approximately 14% and the talc group at approximately 12%. By day 14, the granite-and talc-exposed groups showed particle ingestion by < 10% of cells. Within the talc-exposed group, there was a significant difference in PMN ingestion of particles between days 1 and 14 and days 4 and 14. There was also a significant difference between the talc- and granite-exposed groups on day 14. Supplemental Fig. 1 illustrates high magnification images of birefringent talc particles in PMN.

Figure 4c shows the % of giant cells in the control-, talc-, and granite-exposed groups. The control group had 0% giant cells across the 14 days. The granite group also showed very low levels of giant cells. The talc group had less than 1% giant cells on day 1, which increased to 3% by day 4. By day 7 and 14, the percentage of giant cells remained at about 2%. The number of giant cells in the granite group was not statistically significant compared to the control, or within the group itself across the 14-day study. There was no significant difference in % giant cells when comparing the levels on each day of assessment in the talc-exposed group. There were significant differences (*p* < 0.05) between both the control and the talc-exposed groups, and between the talc- and granite-exposed groups on day 1, 4, 7, and 14.

Figures 5 and 6 quantify the % of cells with ≤3 and > 3 particles/cell over the 14-day study. Figure 5 shows the % of cells with associated particle uptake in the macrophages of the talc- and granite-exposed groups with ≤3 cell-associated particles. On each day, there were more cells with ≤3 talc particles than cells with ≤3 birefringent granite particles, and this difference was significant overall between the talc- and granite-exposed groups by ANOVA analysis. At each time point, the difference was also significant (*p* < 0.01 or greater at all time points), i.e. there were more cells with small numbers of particles in the talc-exposed compared to the granite-exposed animals. Figure 6 shows the percentage of cells with associated particle phagocytosis in macrophages with > 3 particles. On day 1, the granite-exposed group had 0% of macrophages with > 3 particles. The talc-exposed group had almost 10% of macrophages with > 3 particles. This difference was statistically significant (*p* = 0.02). On day 4, 7, and 14, the granite group had more macrophages with > 3 particles than the talc-exposed group. The uptake differences for > 3 particles on days 1, 7, and 14 were statistically significant (*p* = 0.02, *p* = 0.0001, and *p* = 0.02 respectively), but only on day 4 were the differences between the talc- and granite-exposed groups not significantly different.

## Discussion

SEM/EDX findings for talc used in the original study (Beck et al. [15]) were not presented in detail, but the authors made descriptive statements to which our findings can be compared. Our measurements of the toxicity of talc-containing dust and its effect on inflammatory cells were generally consistent with the original Beck et al. [15] study. No asbestos fibers were found, but we did find that 22% of the instilled talc-containing sample met morphologic criteria for fibers, with overall 15% of the material instilled meeting the morphologic and compositional criteria for fibrous talc. In a study to determine the effects of fibrous talc on Hamster Tracheal Epithelial (HTE) cell colonies, colony number decreased as a sign of cytotoxicity [18]. This study was conducted using fibrous talc that had no detectable asbestos, thus providing evidence that fibrous talc per se has inherent toxicity. There have been concerns, cited in the literature, that long-term exposure to talc (both fibrous and not) may be carcinogenic. Studies of Yamada et al. [19] suggested a correlation between fibrous talc and female lung cancer. A recent study suggested a correlation between talc exposure and lung cancer regardless of whether asbestos is present at detectable levels [20].

The International Agency for Research on Cancer (IARC) lists talc containing asbestiform fibers (defined by IARC as talc forming mineral fibers that are asbestiform in their mineral “habit”, *not* talc containing asbestos) as a class I carcinogen [21]. The term asbestiform was defined mineralogically and discussed in detail as it relates to silicates by Virta [22]. Animal studies supporting the toxicity of asbestos-free talc indicate that talc is cytotoxic to macrophages, and talc may induce fibrosis and chronic inflammation [23].

In the Beck et al. [15] study, it was reported that quartz accounted for < 1% of their talc dust composition. In our re-analysis of their material, we found no particles with spectra consistent with silicon dioxide or alpha quartz. Because quartz is a known cause of pulmonary inflammation, toxicity, and fibrogenesis, its absence here is significant and indicates that the pathogenicity of talc in our study cannot be explained by quartz contamination of the dust instilled.

Particles designated as talc by SEM/EDX in this report were required to meet the criterion of Mg/Si atomic weight % ratio being within ±2σ of the mean value, based on the gaussian distribution of this ratio found in our past EDX studies of various talc samples [24]. Previously published studies from our laboratory have used the criterion of being within ±5% of the theoretical atomic weight Mg/Si % ratio (0.649) for the diagnostic identification of talc in human tissues, which compares favorably to a ± 2σ standard [24–26]. The ±5% criterion is conservative, especially when assessing micron-sized unknown particles in human tissues, but it assures the identification of talc and limits the possibility of false positive or false negative errors [24]. Magnesium silicates with traces of other elements commonly found with talc were found in the samples analyzed in our study. Such materials were classified as non-talc magnesium silicates, and is consistent with the fact that such silicates are commonly found within the Earth’s crust. Trace amounts of aluminum, iron, and calcium may be found in talc [27], and these could result in the Mg/Si atomic weight % ratio falling outside the criterion for talc previously described. Traces of magnesium calcium silicates, magnesium titanium silicates, and magnesium aluminum silicates were found in the talc sample as well. These silicates are considered relatively non-toxic, and some have been shown to only cause pulmonary damage when inhaled in large amounts [17].

A chromium-containing particle was found in our analysis. Chromium is a known contaminant of talc, but the relevance of a single such particle to the inflammatory parameters in our study is uncertain. Chromium is a toxic element [28], but more chromium would need to be found to allow a fair assessment of toxic contribution. Iron has the potential to generate reactive oxygen species via Fenton Chemistry and hence may contribute to toxicity [29]. In our study, there were trace amounts of iron with some talc particles. Iron in more substantial amounts has also been shown to contribute to the carcinogenicity of asbestos [29]. There is no convincing evidence that trace amounts of iron in talc significantly contribute to toxicity. In our current study, we were not able to analyze the granite dust from the original Beck et al. [15] study by SEM/EDX, since unlike the talc dust, it was no longer available.

By using polarized light microscopy in this study, we were able to quantify the number and size of particles associated with BAL cells. In the Beck et al. 1987 study [15] as shown in Table 1, at one day after talc instillation, macrophage numbers did not increase, but PMNs migrated into the lung with almost twice as many PMNs after granite exposure than talc at the highest dose. This suggests that the presence of talc particles was sufficient to induce an inflammatory response. Our data show (Fig. 3, 4a, b, and a) that one day after exposure, talc in both particulate and fiber forms had been ingested by cells which far exceeded that of granite ingestion. This is a significant finding because granite particles as measured by Beck et al. [15] were considerably smaller than talc particles. Most studies [30–32] show smaller particles are phagocytosed to a greater extent and have more toxicity per unit mass compared to larger particles. Some studies [33] indicate that larger particles may have greater toxicity with some material compositions. However, studies generally do not examine the differences in particle ingestion by cells over time in vivo, while most in vitro studies that make this assessment show no difference in cellular uptake and considerable uptake of particles within hours, regardless of material or particle size (Reviewed by Brain et al. [34]). Therefore, it is unlikely that the particle size differences between talc and granite particles in this study can explain the difference in cellular uptake of talc and granite on day 1.

Data from the Beck et al. [15] paper suggest that talc and granite may have different toxic mechanisms. Granite induced more PMNs into the lung initially, and initially inhibited the lambda assay (a measure of phagocytic ability) 1 day after instillation. However, over time (day 4 after instillation and later), phagocytic ability with granite exposure returned to control levels. Also, PMNs eventually returned to control levels with granite instillation. However, importantly with talc exposure, PMN numbers were not as high as with granite exposure, but never returned to control levels. Similarly, phagocytic ability with talc (as shown by the lambda assay) remained depressed throughout the 14 days of observation. Our studies follow that same pattern with cellular uptake of talc being less than granite throughout the study except for day 1. Taken together, these observations suggest there may be subacute levels of persistent toxicity with talc exposure. The decrease in phagocytosis by macrophages and PMNs and the increase in giant cells for the talc-exposed group may be due to particle toxicity and the mechanisms by which talc particles inhibit phagocytosis.

There was relatively little birefringent fiber ingestion overall in the exposed talc group and none at all in the exposed granite groups (Fig. 3b). There were no fibers found in the samples from the animals exposed to granite, but as previously mentioned, the granite dust from Beck et al.’s [15] original 1987 study could not be analyzed by SEM/EDX and compared. Thus, we do not know if it had any fibrous particles. Clearly, the presence of at least some phagocytosed fibers in the talc-exposed animal group, combined with the SEM/EDX data of the talc dust, indicates that talc fibers were indeed instilled and then ingested by cells.

The patterns in birefringent particle cellular ingestion between Fig. 3, 4a and a (data involving macrophages and other cells) are consistent with the inflammatory response that would be expected. Macrophages are the main cell involved in the early inflammatory response, and the quicker and larger macrophage initial uptake of talc raises the possibility that talc binds more quickly to the scavenger receptors on macrophages, or that talc particles may bind opsins better and thus have more potential receptors available for particle uptake. Finally, talc may have intrinsic qualities as a stimulant of phagocytosis, which merit further investigation. Recent studies of McDonald et al. [25] of talc distribution in the resected tissues from ovarian cancer patients show macrophages in various pelvic tissues in association with very large numbers of intracellular talc particles.

Could our observation of overall decreased macrophage phagocytosis of talc, of decreased uptake of radioactive particles in Beck’s lambda assay across the time course of that study, and the persistence of neutrophils in the lung be a manifestation of particle overload rather than inherent toxicity of talc? Particle overload is caused by very high concentrations of inhaled particles (reviewed by Brain et al. [34]) and is a common phenomenon seen in rodent animal studies when particle doses are high [35]. Studies have shown that macrophages are able to ingest most amounts of particles in the first 24 h, unless the macrophage becomes overwhelmed by the volume of ingested particles [36, 37]. However, in this study, intracellular and extracellular birefringent particles, as can be seen in the pictures in Fig. 2, tend to be smaller micron-sized particles, and do not appear to reach the average overload volume required for this to be a significant mechanism in the study. Thus, we believe that the intrinsic toxicity of the particles and the phagocytic process with resultant mediators that follow from this process are the most likely explanation for the inflammatory response to both talc and granite.

Since hamsters with intratracheal instillation are the model used in this study, it is important to consider the merits of this model in regard to humans and other species as well as intratracheal instillation versus aerosol exposure. Intratracheal instillation tends to produce different lung distribution patterns compared to inhalation [38]. However, intratracheal instillation has numerous laboratory advantages, including more facile setup, considerably less test material required, less risk to laboratory personnel, and focused exposure into the animal’s lungs. Instillation tends to result in a more centralized pulmonary distribution of deposition (the degree of which is influenced by the volume of carrier fluid), and a more basilar deposition of particles compared to aerosols which tend to be more apical [38]. These differences tend to be more relevant in the interpretation of some dose related outcomes of experimental studies of pulmonary exposures. Hamsters, as a model, had been regularly used to test carcinogens in the development of experimental lung cancers [39]. Hamsters, compared to rats, appeared to be more susceptible to fiber-induced mesothelioma, but less susceptible to the induction of lung tumors with instillation of carcinogens (Reviewed by Brain et al. [34]). Hamsters are noted to have a higher rate of particle clearance compared to humans and to have the greatest phagocytic ability among respiratory animal models (Reviewed by Brain et al. [34]). Although only male hamsters were used in this study, a recent study suggests that macrophage responses, especially phagocytic capability, may be more robust in female hamsters compared to males [40]. Examination of possible sex differences in hamster lung inflammatory responses to environmental particles would be an interesting future study.

The large amount of talc particle ingestion by neutrophils (Fig. 4b) is consistent with talc’s potential ability as a chemoattractant and a phagocytic stimulant. The birefringent particle ingestion rate of talc by neutrophils was much higher on day 1 compared to the comparable value for granite. In our study, talc uptake exceeded granite uptake by neutrophils on most days of analysis and was statistically significant at two of the four studied time points. Neutrophils tend to respond in the first 1–3 days as part of the generalized inflammatory response, which may be why their phagocytic activity was high at that stage.

The dosages of both talc and granite ranged from 0.5 to 2.5% instillation. In the Beck et al. [15] 1987 study, as the dosages for the treated granite group increased, so did the number of neutrophils. With talc exposures, an increase in neutrophils was seen through moderate dosages, but then neutrophils did not further increase at the maximum dose. Since macrophages signal the influx of neutrophils via cytokines and chemokines [41], it is likely that this macrophage function is also diminished by talc exposure in unison with other aspects of macrophage function as noted. Beck et al.’s [15] data regarding the time course of neutrophil influx for talc vs. granite suggest partial recovery of the signaling function over time after talc exposure so that neutrophils, which have a very short tissue life span [42],may continue to infiltrate the lung from the blood. We found significantly greater ingestion of birefringent particles by neutrophils with talc exposure compared to granite at days 1 and 14, but not on day 4.

The frequent finding of PMNs with cytoplasmically located, apparently phagocytosed exogenous particles in both the talc- and granite-exposed groups, at multiple time points, was an interesting observation. PMNs are well known to have phagocytic properties in the context of defense against microorganisms [43]. PMNs are capable of forming a complex known as extracellular trap (NET) in response to various categories of infectious stimuli and small exogenous particles, including silica [44]. In this process, extracellular DNA and chromatin is released from the PMN along with a complex of histones and antimicrobial proteins; however, the PMN undergoes necrosis as a consequence and it is not typically an internal phagocytic process [44, 45]. In a study of the physiology of phagocytosis, it was shown that human PMNs could phagocytize polystyrene beads through an Fc-antibody-mediated binding process [46]. In short, our PMN morphological findings are not entirely with precedent in the literature and suggest that these cells’ phagocytic function against exogenous particulates may be more common than believed. The similarity of PMN phagocytosis with that of macrophages, as well as the roughly similar courses of uptake and retention within the two cellular categories (faster uptake with talc, longer retention with granite, Fig. 4a and b), is also notable. Ours was an animal (hamster) model, and the aforementioned PMN studies derived from humans, so potential cross-species differences should be regarded as a caveat.

The data from Fig. 4c showing marked persistence of giant cell inflammation in talc-exposed animals compared to granite-exposed animals is significant: Giant cells have a tendency to form when the particles are too large to be phagocytosed by individual macrophages or when an inflammatory stimulus is unusually durable and/or persistent. This could signify that talc particles were larger, more durable, and/or less easily phagocytosed, making it more likely for giant cells to form [47]. The studies of Beck et al. [15] did not include data on giant cells.

There were a large number of macrophages with ≤3 talc particles (Fig. 5) and a large number of macrophages with > 3 granite particles (Fig. 6). This could be an indicator of an earlier inflammatory response due to stimulation by the talc particles compared to the granite particles. The talc, which caused an earlier inflammatory response, apparently hindered the macrophages from phagocytizing more talc particles. This mechanism did not appear to be a feature of granite particles, which had a later response. Beck et al. [15] noted that the talc particles seen had a mass diameter that was larger than the granite particles. Seen in Fig. 5 of our study, the macrophages phagocytosed more talc particles at a low intracellular level (< 3) compared to granite particles. The lack of macrophages containing > 3 cell-associated talc particles could be due to the rapid elimination response for such macrophages [48]. This is consistent with earlier observations and discussion that talc, as a whole, may be more toxic to macrophages than granite.

Our study regarding talc and inflammation has relevance to the major current health concern for talc: the previously mentioned association between perineal talc use and ovarian carcinoma [1–5]. This is true given the in vitro demonstration of talc’s pro-inflammatory effects on epithelial ovarian carcinoma cells, and the concomitant effects on point mutational activity, increased cell proliferation, and decreased apoptosis [49], and the well-known observation that macrophages play an important response in mediating inflammatory effects and cellular damage [50]. Also relevant to the current study’s findings regarding macrophages are the various inflammatory infiltrates, including macrophages and other cells, that have been seen associated with talc in the surgically resected tissues from human patients exposed to perineal talc [25].

## Conclusions

In general, the results of our study support the findings and conclusions of the earlier Beck et al. [15] study. A key feature and strength of this current study was the good fortune of having their original talc dust and BAL slides still available and preserved for us to analyze, three decades later. In particular, our re-analysis study showed pro-inflammatory and toxic effects of the talc dust on the abilities of macrophages to ingest talc. With SEM/EDX analysis of the talc dust from the original Beck et al. [15] study, we showed that asbestos fibers, silica, and toxic heavy metals could not account for the dust’s pro-inflammatory response or pathogenic potential, simply because they were generally absent in detectable quantities. But by showing a relatively small, but easily measurable, component of fibrous (non-asbestos) talc (15%) in the dust, the data suggest that the fibrous morphology of some of the material might be enhancing the toxic effects.

PMNs are not commonly known to phagocytose environmental particles. Compared to macrophages, PMNs more commonly ingest bacteria by phagocytosis. Macrophages are more commonly known to ingest large or small particles in the body. This study found PMNs phagocytosed both talc and granite particles (Fig. 2), which is not usually reported.

Our results add to the general understanding of talc toxicity (i.e., even without contaminating asbestos) as an important cause of inflammation and cellular functional deficits in the pulmonary setting. This may in turn help inform talc’s role in other areas such as pelvic tissue including the aforementioned relevance of this study to the association between human perineal use of talc and ovarian cancer. In concert with various other published reports that assess environmental exposures, our study also demonstrates the importance of SEM/EDX analysis in the full assessment of the composition, morphology, and pathogenicity of toxic dust exposures.

## Supplementary information

**Additional file 1: Table S1.** Mean Number of Cells Assessed per slide with each treatment on each day of analysis

**Additional file 2: Figure S1.** Talc particles in neutrophils (1 day after each exposure). Each picture has a neutrophil with birefringent talc particles, lymphocyte, and a red cell. Original magnification is 1000X.

## Data Availability

Data and materials are available on request through contact with the corresponding author.
