# Supplementary material for: Analysis of particles from hamster lungs following pulmonary talc exposures: implications for pathogenicity
Source: Part Fibre Toxicol. 2020 Jun 4;17:20. doi: 10.1186/s12989-020-00356-0 (PMC7271432; doi:10.1186/s12989-020-00356-0)
Supplement: Supplementary file 1 — Additional file 1: Table S1. Mean Number of Cells Assessed per slide with each treatment on each day of analysis [file 12989_2020_356_MOESM1_ESM.docx]

Supplementary Table 1.

**Mean Number of Cells Assessed per slide with each treatment on each day of analysis**

| Days | Talc | Granite | Control |
| --- | --- | --- | --- |
| After IT Dust | Mean Number of Cells Assessed per slide | Mean Number of Cells Assessed per slide | Mean Number of Cells Assessed per slide |
| Day 1 | 576 | 771 | 138 |
| Day 4 | 336 | 378 | 127 |
| Day 7 | 255 | 261 | 225 |
| Day 14 | 496 | 225 | 139 |

Note: Ten high power microscopic fields without artifacts were randomly chosen for analysis. Variations in the numbers of cells counted were because of different cell densities on the slides. Figures 3 through 6 in the paper are presentations of various cell types with cell-associated birefringent particles.
