# Supplementary figures and images for: Analysis of particles from hamster lungs following pulmonary talc exposures: implications for pathogenicity
Source: Part Fibre Toxicol. 2020 Jun 4;17:20. doi: 10.1186/s12989-020-00356-0 (PMC7271432; doi:10.1186/s12989-020-00356-0)

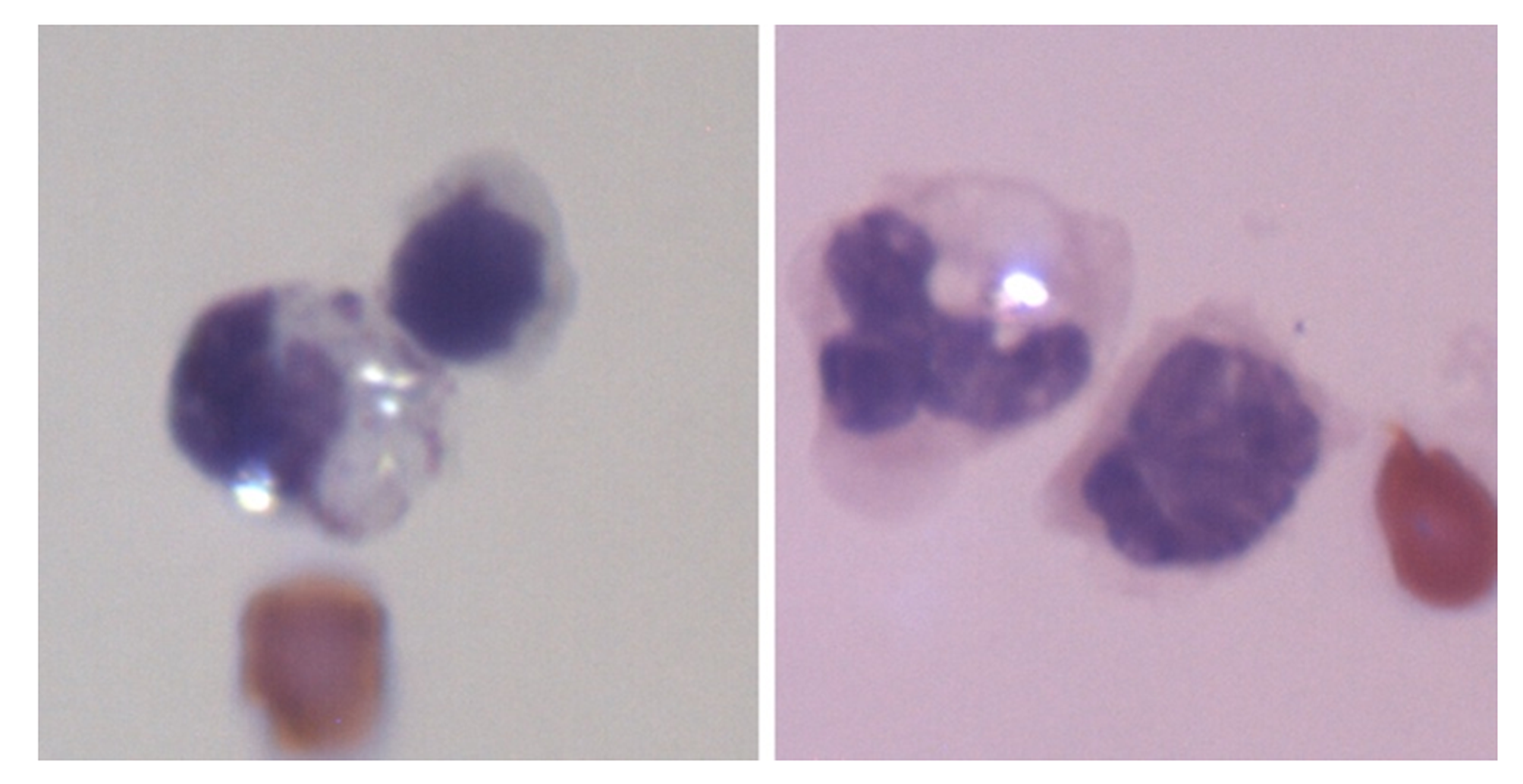

Supplement: Supplementary file 2 — Additional file 2: Figure S1. Talc particles in neutrophils (1 day after each exposure). Each picture has a neutrophil with birefringent talc particles, lymphocyte, and a red cell. Original magnification is 1000X. [file 12989_2020_356_MOESM2_ESM.tif]
